# A Critical Analysis of the FDA’s Omics-Driven Pharmacodynamic Biomarkers to Establish Biosimilarity

**DOI:** 10.3390/ph16111556

**Published:** 2023-11-02

**Authors:** Sarfaraz K. Niazi

**Affiliations:** Department of Pharmaceutical Sciences, College of Pharmacy, University of Illinois, Chicago, IL 60612, USA; niazi@niazi.com; Tel.: +1-312-297-0000

**Keywords:** FDA, omics technology, pharmacodynamic biomarkers, biosimilars, proteomics, glycomics, receptor binding, pharmacokinetics

## Abstract

Demonstrating biosimilarity entails comprehensive analytical assessment, clinical pharmacology profiling, and efficacy testing in patients for at least one medical indication, as required by the U.S. Biologics Price Competition and Innovation Act (BPCIA). The efficacy testing can be waived if the drug has known pharmacodynamic (PD) markers, leaving most therapeutic proteins out of this concession. To overcome this, the FDA suggests that biosimilar developers discover PD biomarkers using omics technologies such as proteomics, glycomics, transcriptomics, genomics, epigenomics, and metabolomics. This approach is redundant since the mode-action-action biomarkers of approved therapeutic proteins are already available, as compiled in this paper for the first time. Other potential biomarkers are receptor binding and pharmacokinetic profiling, which can be made more relevant to ensure biosimilarity without requiring biosimilar developers to conduct extensive research, for which they are rarely qualified.

## 1. Introduction

**Figure 1:**
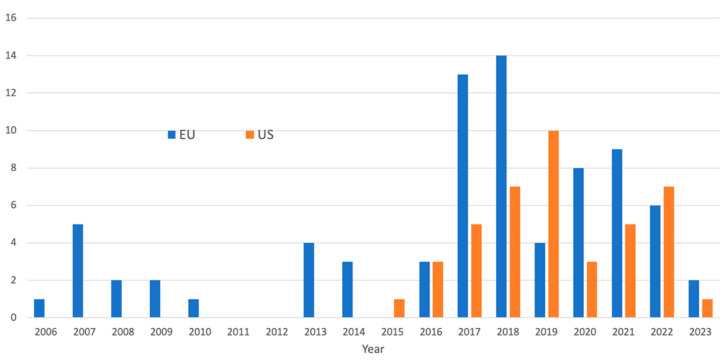
The number of biosimilars approved in the EU and US (as of June 2023) shows a downward trend (source: FDA and EMA).

**Figure 2:**
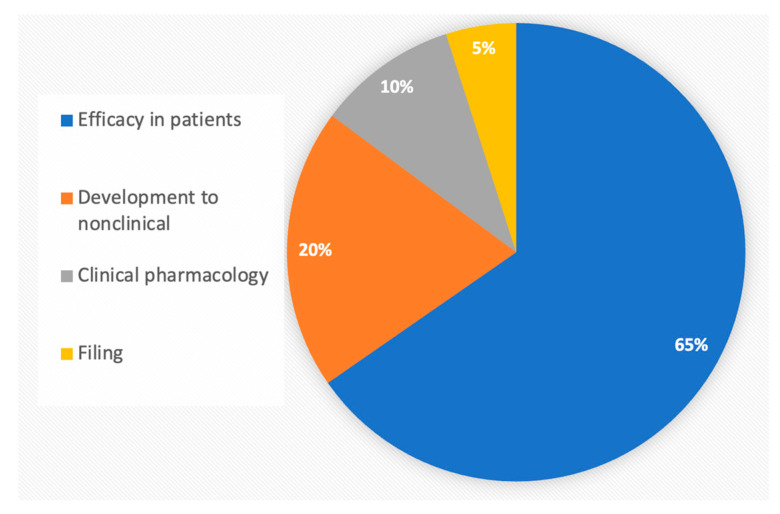
Cost distribution of testing of 246 biosimilars approved in the US, EU, and Japan from 2006 to 2021 [3].

Biosimilars require more extensive testing than generic chemical drugs, owing to the structural variability of recombinant proteins resulting from innate in vivo translation variations. As of August 2023, 42 biosimilars were approved in the United States of America (US), and 74 were approved in the European Union (EU), accounting for approximately 11 molecules and 18 in the EU out of 266 FDA-licensed choices, the majority of which are off-patent [1]. The higher number of approvals in the EU comes from a longer time and the classification of peptides as proteins in the EU. Despite much anticipation, the current downward trend in approving biosimilars (Figure 1) [2] is alarming and is attributed to the high development cost, predominantly towards clinical efficacy testing (Figure 2).

When the European Medicines Agency (EMA) and the United States Food and Drug Administration (FDA) issued their first guidelines, biosimilars were treated as new biological drugs. They were expected to demonstrate clinical efficacy and safety. With the availability of new data, these guidelines have undergone many revisions, reducing, or eliminating testing where justified [4]. In 2005, the FDA withdrew its pivotal guideline, “Statistical Approaches to Evaluate Analytical Similarity” [5] and replaced it with another guideline that has reduced stringency in testing critical quality attributes [6]. In 2019, the FDA issued guidelines suggesting that the immunogenicity testing of biosimilars is unnecessary if differences in immune responses do not alter the pharmacokinetic (PK) profile [7], specifying that in silico approaches can be used to establish immunogenicity profiles [8]. In 2023, an amendment to the US Biological Products Competition and Innovation Act (BPCIA) replaced the term “animal toxicology” with “nonclinical” testing [9,10]. While it is not the purview of regulatory agencies to amend guidelines for reducing developmental costs, their responsibility is to avoid unnecessary exposure to humans [11].

However, one major hurdle in rationalizing the regulatory pathway for biosimilars comes from the requirements mandated in the following statute governing the BPCIA:

*“(cc) a clinical study or studies (including the assessment of immunogenicity and pharmacokinetics or pharmacodynamics) that are sufficient to demonstrate safety, purity, and potency in 1 or more appropriate conditions of use for which the reference product is licensed and intended to be used and for which licensure is sought for the biological product.”* [12]

While the FDA guidelines have suggested that if residual uncertainty exists after analytical and clinical pharmacology assessments, “additional clinical studies” may be required, whether additional studies indicate efficacy testing in patients is still unclear. This could refer to further clinical pharmacology studies, but this perception is clouded by mentioning “…in 1 or more appropriate conditions of use”; this could only mean testing in patients, which is considerably less sensitive in differentiating a biosimilar candidate from its reference product [13].

This misconception has generally led to the clinical efficacy testing of biosimilars in patients, enrolling a median of 538 participants (interquartile range, 372–644 patients) at a median cost of USD 27.6 million each (USD 18.0 million–USD 36.7 million), with an average price per enrollee of approximately USD 55,000. Moreover, these trials last a median of 55 weeks (46–78 weeks) [14]. Oncology drugs have the highest testing costs. More complex trial protocols take longer to design, obtain approval for (institutional and FDA), recruit patients from contracted providers, analyze the resulting data, and submit the results.

As of April 2023, 94,910 participants had been enrolled in 170 active or completed phase 3 biosimilar trials with study sizes ranging from 3 to 4994 participants; among these, 100 studies were marked for cancer (26, 34, 25, 21, and 16 studies for lymphoma, breast cancer, metastatic, HER2, and adenocarcinoma, respectively), 18 for macular degeneration, 31 for rheumatoid arthritis, 24 for psoriasis, and 17 for osteoporosis. All completed trials met the equivalence criteria [15]. Current studies cost more than USD 5 billion based on the average cost per enrollee. Reducing these costs may significantly impact the affordability of biosimilars.

To justify this stage of the development process, the FDA has already proposed that under specific conditions, clinical pharmacokinetic (PK) and pharmacodynamic (PD) data that establish comparable exposure and response between a proposed biosimilar product and the reference product may be satisfactory for a comprehensive evaluation of any clinically significant distinctions between the products. This is despite the requirement for a thorough assessment of immunogenicity [16]. However, this concession excludes most biological drugs, such as monoclonal antibodies, which do not exhibit traditional pharmacodynamic (PD) responses.

In 2018, the FDA embarked on a scientific plan to simplify the approval process for biosimilars under the Biosimilars Action Plan [17]. The primary element of this plan involved the creation of information resources and development tools aimed at aiding biosimilar sponsors in the production of biosimilar and interchangeable goods of superior quality, utilizing cutting-edge techniques. Furthermore, the commitment letter for Biosimilar User Fee Amendments III explicitly addresses the inclusion of PD biomarker utilization as a component of the regulatory science pilot program [18].

In September 2022, the FDA organized its first program to expedite biosimilars’ entry, “FDA Workshop: Increasing the Efficiency of Biosimilar Development Programs” [19]. A subsequent significant change was detailed in a publication by the FDA’s Division of Applied Regulatory Science (DARS) [20], which recommended waiving clinical efficacy testing [21] for molecules with prominent PD biomarkers, which need not correlate with clinical efficacy [22]. Examples include the absolute neutrophil count area under the effect–time curve, which is a more reliable endpoint than the clinical-efficacy endpoint for the duration of severe neutropenia [23].

The DARS made these conclusions based on investigations [24] and clinical studies [25,26,27] to identify the best practices for characterizing PD biomarkers for various drug classes. These studies evaluated the use of human plasma proteomic and transcriptomic analyses to identify novel biomarkers that could be used to secure a waiver for efficacy testing in patients [28].

The FDA has also suggested that PD biomarkers can be identified using technologies such as large-scale proteomic approaches [29], which are not readily available. The FDA has also confirmed that PD biomarkers need not correlate with a clinical response to allow their use for establishing biosimilarity. This conclusion is based on the understanding that similar PD responses lead to identical efficacy responses.

In September 2023 [30], the FDA held a workshop with major regulatory agencies to discuss how patient efficacy testing requirements can be rationalized and harmonized. One regulatory agency, the MHRA, clearly declared that no such testing is required; others suggested that the developers can present arguments to seek such waivers.

## 2. Understanding Pharmacodynamic Biomarkers

A pharmacodynamic marker is a measurable biochemical, physiological, or molecular variable that provides information on a drug’s mechanism of action, efficacy, and safety. In drug development and medical research, pharmacodynamic markers help understand how a drug affects a target organism, which can be at the cellular, tissue, or systemic level.

Without a comparative clinical efficacy study, the FDA guidance documents outline how biosimilars may be approved based on pharmacokinetic (PK) and PD biomarker data. Reliance on PK and PD data allows for shorter and less costly clinical studies that can often be conducted in healthy participants. The PD biomarkers are indicators of a drug’s pharmacological effect on its target or targets. For example, the target might be a receptor molecule that initiates a complex signaling cascade. Changes in the levels of proteins along the signaling cascade or modifications to them could be considered pharmacodynamic responses. Therefore, these proteins could be regarded as PD biomarkers and used to help establish biosimilarity.

Pharmacodynamic markers are crucial for various phases of drug development, including:

- Dose determination—they can help determine the optimal dosage of a new drug by showing its effects at different concentrations;
- Mechanism of action—understanding the changes in pharmacodynamic markers can help elucidate how a drug exerts its therapeutic or adverse effects;
- Efficacy—these markers can provide early indications of a drug’s effectiveness, often before clinical endpoints can be measured;
- Safety—monitoring pharmacodynamic markers can give insights into potential side effects or toxicities, enabling researchers to make informed decisions during clinical trials;
- Personalized medicine—in some cases, pharmacodynamic markers can also help in patient stratification, identifying which subgroups of patients are most likely to benefit from a particular treatment.

A functional PD marker is a specific type of biomarker that reveals a drug’s biochemical or physiological effects on its target or a downstream pathway. Unlike general biomarkers, that might indicate the presence or risk of a disease, a functional PD marker specifically illustrates the drug’s mechanism of action in the body and how the body reacts to it.

PD biomarkers, like hematocrit or WBC count, are pharmacological markers. Since the FDA does not require a correlation between the PD biomarker and the clinical response, a significant issue is created; if a product meets the biomarker profile, does this mean it will also yield the same clinical response? The answer is no; it has been established. Additionally, if a product fails to meet the biomarker profile due to the nonlinearity of the marker vs. the PK profile, does this mean that a product is not equivalent? The answer is no; it has not been established. Both possibilities lead to the limited utility of newly discovered biomarkers but do not apply to pharmacological markers related to response.

PD biomarkers indicate the effects of a drug on its intended target or a downstream pathway. In simpler terms, they give insights into how the drug works in the body and how the body responds to the drug. The term “functional,” in the context of a PD marker, usually suggests that the marker has a direct link or relevance to the drug’s therapeutic response or mechanism of action, as shown in Table 1.

## 3. Omics Technologies

Omics technologies refer to the comprehensive, high-throughput characterization of biological molecules in an organism, cell, or tissue. Genomics is the most widely known “omics” technology, which studies an organism’s entire set of genes. However, there are several other “omics” technologies, each focusing on different types of molecules. Omics studies can be broadly categorized into targeted and untargeted approaches. (Table 2).

The omics technologies compare chemical profiles to identify, characterize, and profile peptides, proteins, glycans, lipids, and other unknown entities resulting from administering a therapeutic protein. The targeted approaches in omics applications involve the analysis of a pre-defined set of molecules or pathways. The researcher knows in advance which specific molecules or features they are interested in, such as targeted genomics (PCR assays for gene sequences) and targeted proteomics (multiple reaction monitoring (MRM), where specific proteins or peptides of interest are quantified). Targeted metabolomics [71] focuses on a particular class of metabolites, such as lipidomics, using methods like selected reaction monitoring (SRM).

The untargeted approaches broadly analyze as many molecules as possible in a sample without a pre-defined focus. The aim is often discovery or to obtain a global overview of a system, for example, untargeted genomics (whole genome sequencing), untargeted transcriptomics (RNA sequencing which quantifies all mRNA transcripts in a sample), untargeted proteomics (mass-spectrometry-based approaches like shotgun proteomics whereas many proteins as possible are identified and quantified), and untargeted metabolomics (high-resolution mass spectrometry or nuclear magnetic resonance (NMR) spectroscopy, aiming to detect as many metabolites in a sample as possible). Table 3 lists the common analytical technologies used in omics technology applications.

### Proteomics

Proteomics is the most relevant application for characterizing and quantifying biomarkers based on the action of proteins as functional molecules in the cell: performing most of the cellular functions and contributing to most of the cell’s structure. Proteomics aims to identify a chemical, such as a protein, carbohydrate, or another entity, which becomes evident only when the reference product is administered compared to a placebo. Detecting these chemical entities requires sophisticated technologies, as listed in Table 4, along with examples of these technologies used to characterize proteins, unknown proteins, and unusual protein structures [57,142].

The technologies for proteomics research, especially the nano-LC and mass spectrometry, have experienced unprecedented development; currently, the proteome of the single cell can be identified in its totality, despite many shortcomings that are fast resolved, leaving a great future for proteomics in drug development; whether they are suitable for biosimilars is another matter that is discussed later in the article.

## 4. Glycomics

Glycomics is a comprehensive study of all glycan structures (sugars and carbohydrates) in each cell type or organism, including the identity of individual glycans and the overall glycan profile. Glycans are crucial components of various biological systems. They play roles in numerous biological processes, including cell–cell communication, immune responses, infection, inflammation, and cancer progression. Glycans are often added post-translationally to proteins and lipids, altering their functions and properties. They also play a critical role in protein folding and stability [154,155]. These patterns can affect therapeutic proteins’ stability, solubility, and biological activity. Understanding glycosylation is vital for developing, producing, and approving biosimilars because even slight variations in glycosylation can result in different clinical outcomes. The evaluation of glycosylation is the second most frequently applied ‘omics’ technology to compare molecules for biosimilarity.

Table 5 lists common analytical approaches to identify and qualify glycans.

Understanding the complex structures of glycans is essential for understanding glycomics. Techniques such as MS and nuclear magnetic resonance (NMR) have been used for this purpose. Glycans often bind to proteins, affecting their functions. Studying these interactions can provide insights into numerous biological processes and diseases to learn how cells synthesize glycans and how these structures contribute to cellular functions and broader physiological processes. Changes in glycosylation patterns are often associated with diseases, including cancer, making them potential biomarkers for disease diagnosis and progression. The glycan profile also establishes high similarity for protein function, stability, and efficacy.

## 5. Transcriptomics

Metabolomics and transcriptomics are branches within systems biology, focusing on comprehensive analyses of biological molecules in an organism, cell, or tissue. However, they target different levels of the cellular molecular hierarchy. Metabolomics studies the complete set of small-molecule metabolites within a biological sample. Its primary focus is on metabolites, including amino acids, sugars, lipids, and other small molecules that are products or intermediates of cellular metabolism [167].

Transcriptomics involves studying the complete set of RNA transcripts produced by the genome under specific circumstances. Its primary focus is messenger RNA (mRNA), but other non-coding RNAs can also be studied. It provides insights into the gene expression patterns, revealing which genes are upregulated or downregulated in particular conditions [168].

Transcriptomics is the scientific discipline investigating the comprehensive collection of RNA transcripts generated by the genetic material (genome) of a cell, tissue, or organism during distinct circumstances or at stages of development. RNA transcripts, encompassing various types such as mRNA, rRNA, tRNA, and non-coding RNA, serve as intermediaries in transmitting genetic information from DNA to proteins. The transcriptome composition exhibits variability based on cell type, developmental stage, environmental circumstances, or pathological conditions. Hence, it is imperative to comprehend the transcriptome to interpret the functional components of the genome, unveil the molecular constituents of cells and tissues, and gain insights into the progressions of diseases. Transcriptomics encompasses many methodologies, such as microarray analysis, RNA sequencing (RNA-Seq), and serial gene expression analyses, employed to investigate gene expression patterns. RNA-Seq has emerged as the predominant technique in current scientific research due to its substantial capacity for high-throughput analysis and comprehensive data acquisition [169] (Table 6).

## 6. Genomics

Genomics is the comprehensive study of an organism’s genes or genomes. It involves sequencing and analysis of genomes’ structure, function, and evolution and provides insights into gene expression, function, regulation, and interactions. Genomics incorporates elements from genetics, but its primary focus is the collective characterization and quantification of genes that direct protein production assisted by enzymes and messenger molecules. Genomic techniques vary from the traditional polymerase chain reaction (PCR) and gene sequencing methods to modern next-generation sequencing (NGS) technologies. These methods have allowed the sequencing of entire genomes, such as in the Human Genome Project, which sequenced the whole human genome and revolutionized biomedical research [179,180].

For recombinant proteins and biosimilars, genomics aids in identifying the genes responsible for desired protein functions, optimizing gene expression, and ensuring quality and consistency in production.

One characteristic feature of eukaryotic aging is the degradation of epigenetic information. This phenomenon can be counteracted through a process known as ectopic induction. In mammals, the Yamanaka factors OCT4, SOX2, and KLF4 (OSK) have been found to restore DNA methylation patterns, transcript profiles, and tissue function associated with youthfulness. Importantly, this restoration occurs without compromising the cellular identity, necessitating the active removal of DNA methylation. High-throughput cell-based assays, such as transcription-based aging clocks and real-time nucleocytoplasmic compartmentalization assays, can differentiate between young and old and senescent cells. These assays can be employed to identify compounds that have the potential to reverse cellular aging and rejuvenate human cells while maintaining the integrity of the genome. Consequently, six chemical cocktails have been identified in less than a week, restoring a youthful genome-wide transcript profile and reversing transcriptomic age without compromising cellular identity; thus, genetics and chemical means can achieve rejuvenation by age reversal [181].

The following are some aspects of the use of genomics:

Structural genomics—this area of genomics involves the characterization and mapping of genomic structures, including the sequencing of whole or significant parts of the genome;

Functional genomics—the study of gene and protein functions and their interactions. The techniques used in functional genomics include transcriptomics (gene expression analysis), proteomics (protein expression analysis), and metabolomics (metabolic profile analysis);

Comparative genomics—comparative genomics involves comparing the genomes of different species to understand the similarities and differences in structure and function. This can be used to infer evolutionary relationships among organisms;

Genomic medicine—the application of genomics in health and disease research can help identify disease-susceptibility genes, develop diagnostic tests, and enable personalized treatment strategies based on a patient’s genetic makeup, often called precision medicine.

The following techniques highlight genomics’ broad and powerful impact on recombinant protein research, including improvements in design, production, stability, and therapeutic applications:

- Whole genome sequencing;
- RNA-Seq—analyzing the quantity and sequences of RNA [53];
- Quantitative PCR—quantitative measurement of specific DNA or RNA levels [181];
- Microarrays—high-throughput gene expression analysis [54];
- Comparative genomic hybridization—detecting and mapping chromosomal imbalances [182].

Combining traditional monitoring techniques with omics technologies represents a unique opportunity to characterize the host cell culture state better and shift from an empirical to a rational approach for process development and optimizing bioreactor cultivation processes. A few examples of genomics applications in the field of recombinant proteins are presented in Table 7.

## 7. Epigenomics

Epigenomics studies a complete set of epigenetic modifications in DNA or associated proteins other than the DNA sequence, termed the epigenome, that cells use to control gene expression. Epigenetic modifications can influence gene expression without altering the underlying DNA sequence. These modifications play critical roles in cell differentiation and disease, and unlike DNA sequences, they can be changed by environmental conditions. Epigenomics often involves NGS technologies such as whole-genome bisulfite sequencing for DNA methylation and chromatin immunoprecipitation sequencing for histone modifications [193,194,195].

The following are the major types of epigenetic modifications:

- DNA methylation—in mammals, DNA methylation typically occurs at cytosine residues in the cytosine-phosphate-guanine context and is an essential process for normal development. Changes in DNA methylation patterns are associated with several key processes, including carcinogenesis;
- Histone modification—histone proteins can be modified post-translationally by methylation, acetylation, and ubiquitination. These modifications can alter the chromatin structure and affect gene expression;
- Studying the extent to which combinations of DNA-, RNA-, and PTM-level variations contribute to the complexity of the human proteome.

The application of epigenomics to recombinant proteins is an evolving area of research. Table 8 lists a few examples of the applications of epigenomics.

## 8. Metabolomics

Metabolomics is the large-scale study of small molecules within cells, biofluids, tissues, or organisms, commonly known as metabolites. These metabolites are the end products of cellular processes and form a significant part of the metabolome, which comprises all metabolites in a biological organism. Metabolomics is a compelling approach in systems biology, as metabolites are often the end products of cellular processes, and changes in their concentrations can be more representative of the current biological status than changes in other biomolecules. Metabolomics is used for various purposes, including studying disease mechanisms, biomarker discovery, drug discovery, and understanding drug effects. This is a critical field in precision medicine and personalized healthcare [202,203,204]. Integrating metabolomics with recombinant protein research offers a multifaceted approach to improving protein production, understanding protein function, and creating more effective therapies. These applications are continually evolving with advancements in analytical techniques and technologies (Table 9).

## 9. FDA Omics Perspective

The FDA recommends using omics technologies to identify PD markers for biologicals that do not possess known PD markers, such as hematocrit content on exposure to erythropoietin or white blood cell count on the administration of filgrastim, to waive efficacy testing in patients. Omics refers to comprehensive testing involving high-throughput, large-scale, and integrative approaches for studying and analyzing various components of biological systems. The major omics technologies include genomics, transcriptomics, proteomics, metabolomics, and epigenomics. The FDA has recently conducted investigations and demonstrated how to apply omics technologies to identify novel PD biomarkers without known biomarkers, which can be used for the similarity assessment of biosimilars to waive the need for efficacy testing in patients [28].

### 9.1. FDA Research

The FDA has undertaken empirical research on biomarkers associated with Parkinson’s disease (PD) to expedite the development of biosimilars [210]. This study encompasses clinical pharmacology investigations wherein participants are administered different biological dosages of a drug, and researchers assess the corresponding biomarker response. The correlation between the dose administered and the response of the biomarker may suggest that the biomarker is suitable for a pharmacodynamic similarity investigation.

The omics exercises can result in functional biomarkers that are part of the mechanism of action, as shown below, or a result of the activity of the administered protein, whether associated or not with the mechanism of action.

#### 9.1.1. PCSK9 Inhibitor Markers (Lipidomic Exercise)

The FDA has approved two cholesterol-lowering drugs, alirocumab, and evolocumab, in a class of biologics known as PCSK9 inhibitors. These inhibitors block PCSK9, which prevents the body from removing excess cholesterol. In an FDA study, 72 healthy participants received different doses of evolocumab (21, 35, 70, or 140 mg), alirocumab (15, 25, 50, or 100 mg), or a placebo [211]. The researchers analyzed two serum biomarkers: low-density lipoprotein cholesterol (LDL-C) and apolipoprotein B (apoB). The primary outcome measures consisted of the areas under the effect curve (AUEC), which quantified the temporal biomarker response and the most significant deviation from baseline values of LDL-C and apoB in serum. The study’s findings exhibited a clear and direct relationship between the dosage of alirocumab and evolocumab and the pharmacodynamics-associated biomarkers. This relationship was evidenced by a substantial alteration in LDL-C and apoB levels compared to the initial measurements as the doses were escalated. Nevertheless, it was shown that apoB demonstrated greater variability in response compared to LDL-C in the study subjects. Therefore, low-density lipoprotein cholesterol (LDL-C) and apolipoprotein B (apoB) have the potential to serve as appropriate biomarkers for comparative investigations of alirocumab and evolocumab in the context of Parkinson’s disease (PD) [212].

#### 9.1.2. IL-5 Antagonists Biomarkers [26] [Functional Markers]

The class of biologics known as anti-IL-5 has been authorized to treat severe eosinophilic asthma, a condition distinguished by an overabundance of eosinophils, a type of white blood cell. Interleukin-5 (IL-5) is the primary cytokine responsible for activating eosinophils, thus leading to airway inflammation. Interleukin-5 (IL-5) antagonists effectively inhibit the activation of eosinophils by preventing IL-5 from binding to its receptors. Three biologics, namely benralizumab, mepolizumab, and reslizumab, have received approval within the IL-5 class of anti-asthma drugs. In a research investigation, a total of 72 individuals who were in good health were randomly assigned to one of two groups. The first group received a placebo, while the second group received a dosage of either mepolizumab (ranging from 3 to 24 mg) or reslizumab (ranging from 0.1 to 0.8 mg/kg), both of which were administered at a lower level than the therapeutic dose. In this investigation, eosinophils in circulation were utilized as biomarkers for Parkinson’s disease (PD). The study’s primary objectives were the highest change observed from the baseline measurement and the area under the effect curve (AUEC). A dose-response association for eosinophil counts was not identified due to the significant heterogeneity in data across the study participants. The administration of the maximum dosage of mepolizumab at 24 mg and reslizumab at 0.8 mg/kg demonstrated observable therapeutic effects. The researchers refrained from doing the intended dose–response analysis due to the significant variability observed in the data. The present work employed pre-existing models and simulations to investigate the impact of various dosages of IL-5 antagonists, including doses up to the therapeutic threshold, on the levels of circulating eosinophils. The findings indicated that administering greater doses, namely therapeutic dosages, might sufficiently discern the distinctions between products using circulating eosinophils as biomarkers in research assessing pharmacodynamic similarities.

#### 9.1.3. IFNβ-1a Biomarkers [213] [Proteomics Markers]

In a particular investigation, the FDA examined the application of proteomics, which involves the comprehensive analysis of proteomes or sets of proteins, to uncover biomarkers for Parkinson’s disease (PD) in biological treatments such as IFNβ-1a and pegylated-IFNβ-1a (pegIFNβ-1a). These products are used to treat multiple sclerosis; however, their mechanisms of action and the relevant PD biomarkers are still unclear, although there are potentially reported candidates. To address this issue, proteomics evaluated circulating biomarkers by assessing more than 7000 proteins from plasma samples to identify potential PD biomarkers. In the present investigation, a total of 84 individuals who were in good health were administered a therapeutic dosage of IFNβ-1a, pegIFNβ-1a, or a placebo. During the preliminary examination, data exclusively from the 36 participants who were administered either a placebo or the highest dosage (30 µg IFNβ-1a or 125 µg pegIFNβ-1a) were assessed. Baseline blood samples were obtained, followed by further collections at many time points. The proteins were evaluated using the proteome test SOMAscan (version 4.1). This investigation successfully identified 248 differentially expressed proteins in the treatment group compared to the placebo group for IFNβ-1a and 528 differentially expressed proteins for pegIFNβ-1a. The researchers prioritized 31 proteins that exhibited the most notable distinctions from the placebo group. Among these proteins, eight had a maximal change from the baseline that exceeded a factor of four. The identification of candidates, both previously reported and newly discovered, was accomplished by researchers. This study showcases the utility of proteomics in identifying potential biomarkers for Parkinson’s disease (PD). These biomarkers can be employed in clinical pharmacology studies to facilitate the creation of biosimilars. This is especially valuable when the products exhibit intricate mechanisms of action or when only a limited number of PD biomarkers have been identified.

#### 9.1.4. Model-based Testing Markers [214] [Functional Markers]

The FDA has also presented results on a model-based approach for the dose selection (MBADS) of pegfilgrastim, which treats neutropenia (low white blood cell count) caused by anticancer medications. Multiple biosimilars have received approval, and to support their approval, pharmacokinetic (PK) and pharmacodynamic (PD) approaches have been employed. These approaches have utilized the biomarker of absolute neutrophil count. Several projects utilized a dosage of 6 mg for pharmacokinetic (PK) and pharmacodynamic (PD) similarity investigations, while alternative programs employed a dosage of 2 mg. Pegfilgrastim exhibits a non-linear pharmacokinetic profile, indicating that the elimination of the drug from the body is influenced by the dosage administered. This factor makes calculating the optimum dose for PK and PD similarity tests more complex—the present work employed model-informed drug development (MIDD) methodologies to address this issue. MIDD approaches employ quantitative methods and data sources to facilitate drug development and regulatory decision-making. In this work, the researchers modified an existing model by incorporating data from two phase I trials. These trials involved administering a single dosage of either 30, 60, or 100 μg/kg of pegfilgrastim to healthy volunteers. The researchers conducted simulations with two doses, specifically 2 and 6 mg. The findings from the simulation analyses indicate that the administration of 6 mg of pegfilgrastim is adequate for detecting distinctions between a proposed pegfilgrastim biosimilar and the reference product in studies examining pharmacokinetic (PK) and pharmacodynamic (PD) similarities. The findings of this study demonstrate that the utilization of simulations can effectively facilitate the process of dose determination for biosimilars exhibiting non-linear pharmacokinetics.

The FDA has demonstrated the use of omics technologies to discover pharmacodynamic markers for drugs like the mAbs that do not demonstrate functional pharmacodynamic biomarkers. To enable applications of the FDA suggestions, we need to review the role of omics technologies, how they are used in the development of new drugs, and their current status, and to analyze whether there is a need to use omics technologies to demonstrate biosimilarity, given the available biomarkers, the difficulties in applying omics technology for the development of biosimilars, the complexities created by individualized omics applications and finally, the scientific rationale of using the omics approach.

### 9.2. The Practicality of Omics Technologies

While the FDA’s suggestions for engaging in omics technologies to identify PD markers are scientifically sound, their application is complex, and it is questionable if this should be the responsibility of developers. The selection of a PD biomarker requires several considerations:

- Relevance to the mechanism of action;
- Sensitivity to differences between the proposed biosimilar and the reference product;
- Analytical validity;
- Time of onset correlated with dosing;
- The dynamic range over the exposure range.

Large-scale proteomic methods allow for simultaneous study of the expression of several proteins after administering the reference product; however, it will always be a random choice, varying among developers. Without a specific criterion to compare one PD marker against another, it will always be uncertain if biosimilarity based on one set of PD markers is more robust than that with another set of PD markers. The same applies to other omics platforms like glycomics, transcriptomics, and metabolomics.

Because developers plan these studies separately, their findings may differ, although all will be relevant. Additionally, these considerations for qualifying biomarkers require extensive testing and validation, which will likely consume more time and investment than conducting efficacy testing on patients, leaving little incentive for developers to engage in omics technology [215].

However, it is inappropriate to expect developers to identify proteomic profiles for several reasons:

- Proteins located remain unidentified, and these may well be testing-process dependent;
- Proteins may not be related to clinical efficacy and only represent a clinical pharmacological profile;
- The expertise of proteomic technology is limited to biosimilar developers who are not necessarily research entities; expecting them to use these technologies may result in conclusions that may be less reliable than the standard technologies.

### 9.3. Biosimilars and Omics Technologies

With their comprehensive and high-resolution data outputs, omics technologies can be crucial in biosimilars’ efficacy testing and characterization (Table 10).

## 10. Omics Alternatives

These PD biomarkers, like hematocrit or WBC count, are pharmacological markers that correlate with clinical response, but the precursors to the pharmacological markers, the PD markers, need not correlate with the clinical response, creating a major issue; if a biosimilar meets the PD biomarker profile, does this mean it is also going to yield the same clinical response? The answer is not necessarily; it has been established. Additionally, if a product fails to meet the biomarker profile due to the nonlinearity of the marker vs. the PK profile, does this mean that a product is not equivalent? The answer is no; it has not been established. These possibilities show the limited utility of newly discovered biomarkers but do not apply to response-related pharmacological markers.

While FDA efforts have introduced many scientific ideas to improve the assessment of biosimilarity, these may not be the most practical, especially when several other more straightforward comparative testing possibilities are available [216,217]. New robust analytical tests, such as MS, can be used to perform orthogonal biosimilarity testing, such as multiple-receptor binding comparisons, as this is the primary mechanism that triggers PD biomarkers (if available).

### 10.1. Receptor Binding

In the cascade of events, before a PD response is triggered, the protein molecule first binds to its receptors, a well-known and established mechanism of action. The clinical response to biological drugs is based on their mechanism of action, which begins with receptor binding. Current science has made this testing highly accurate and objective. mAbs can also interact with multiple receptors and can be evaluated using orthogonal analytical methods. The primary receptors involved in the activity of the therapeutic proteins include (parenthetical entry shows the number of such receptors). The primary receptors for approved protein therapeutics include: glucagon-like peptide 1 receptor (3), insulin receptors (3), heat-stable enterotoxin receptors (2), adrenocorticotropic hormone receptor, angiotensin II type 2 (AT-2) receptor, corticotropin-releasing factor receptor 1, glucagon-like peptide 2 receptor, gonadotropin-releasing hormone receptor, notch signaling pathway, oxytocin receptor, parathyroid hormone receptor, parathyroid hormone/parathyroid hormone-related peptide receptor, prothrombin, receptor tyrosine-protein kinase erbB-2, secretin receptor, somatostatin receptor 2, somatostatin receptor 5, type-1 angiotensin II receptor, vasopressin V1a receptor, vasopressin V1b receptor, vasopressin V1a receptor, vasopressin V1b receptor, vasopressin V2 receptor [218] (Table 11).

An orthogonal approach to establish comparable receptor binding would be substantially more robust and objective than finding an unknown PD marker, qualifying it, and testing it to establish biosimilarity. This powerful test demonstrates functional similarity when PD biomarkers such as mAbs are unavailable.

### 10.2. Pharmacokinetic Profiling

While receptor binding leads to PD response, determining the clinical response, the PK profile is one of the strongest PD- and clinical-biomarker surrogates.

The use of pharmacokinetics (PK) as a surrogate for pharmacodynamic (PD) markers in the development and evaluation of biological drugs is a topic of increasing interest. Traditionally, PK and PD have been viewed as separate, yet interconnected, disciplines: PK focuses on the absorption, distribution, metabolism, and excretion of a drug, while PD investigates the drug’s physiological and biochemical effects [244]. However, numerous studies suggest that PK parameters can serve as surrogate PD markers, particularly in establishing the biosimilarity of a candidate drug to a reference product [244].

For instance, the PK profile provides a comprehensive overview of a drug’s disposition kinetics, often indicating its effectiveness or safety [245]. Key PK parameters like Cmax (peak serum concentration of the drug) and AUC (area under the curve, reflecting overall drug exposure) are frequently employed as surrogate endpoints in clinical studies for biological drugs [246].

In monoclonal antibodies (mAbs), PK measures such as clearance rate and volume of distribution have shown strong correlations with efficacy markers like tumor-size reduction [247]. Likewise, PK parameters are commonly used as surrogates for PD endpoints in other classes of biological drugs, including erythropoiesis-stimulating agents and interferons [248].

Regulatory agencies like the FDA are increasingly recognizing the potential of PK as a surrogate for PD markers. For example, the FDA’s guidance for biosimilars acknowledges that comparative PK and PD data may suffice to demonstrate biosimilarity without additional clinical studies under certain conditions [249].

It is essential, however, to recognize that the utility of PK as a surrogate for PD markers is not universally applicable. Its appropriateness depends on the complexity of the biological drug and the robustness of the PK/PD relationship [250]. Therefore, employing PK as a surrogate for PD markers should be predicated on a comprehensive understanding of both the drug’s mechanism of action and its PK/PD correlation.

The burgeoning body of evidence supports the notion that PK can serve as a viable surrogate for PD markers, streamlining the development of biosimilar and biobetter treatments. For example, PK metrics have been employed as predictors for long-term outcomes in treatments for rheumatoid arthritis [251]. PK parameters like Cmax and Tmax also predict toxicity in specific cancer treatments [252].

Nonetheless, limitations exist. One challenge is the often-unclear relationship between PK variables and complex biological responses, particularly for drugs with intricate mechanisms of action or those used in polytherapy [253].

The volume of distribution (Vd) as a function of time offers another exciting dimension. Unlike in traditional PK, where Vd is often treated as a constant, recognizing Vd as a time-dependent function allows for incorporating factors like tissue perfusion rates, varying clearance rates, and organ-specific uptake or release over time [254]. Such considerations are particularly pertinent for biological drugs, which may exhibit nonlinear kinetics and more complex distribution patterns than small molecule drugs [255].

Several applications have been noted in both pre-clinical and clinical research:

- Cancer chemotherapy—modeling Vd(t) can lead to better predictions of drug concentrations in tumor tissue versus surrounding tissues, potentially optimizing dosing schedules for maximum efficacy and minimal toxicity [256];
- Infectious diseases—understanding Vd(t) can help in designing dosage regimens that ensure sufficient drug concentrations at the infection site while minimizing systemic exposure [257];
- Autoimmune diseases—for monoclonal antibodies used in conditions like rheumatoid arthritis, Vd can change over time due to factors like target-mediated drug disposition. Understanding Vd(t) can inform individualized dosing [258];
- Geriatric pharmacology—age-related physiological changes can impact Vd, and considering Vd as a function of time provides insights into drug disposition in elderly patients [259];
- Drug development—during the pre-clinical phase, understanding Vd(t) can guide decisions about advancing a drug candidate to the next stage, potentially saving time and resources [260].

In conclusion, the role of PK as a surrogate for PD markers is increasingly supported by scientific evidence and accepted in both academic and regulatory settings. While its application depends on various factors, the potential advantages—including accelerated approval processes and reduced development costs—are significant. As the biosimilar landscape evolves, regulatory agencies like the FDA should consider expanding the contexts where PK can be a surrogate for PD markers.

So far, none of those mentioned above technologies—receptor binding, PK profiling, or the long list of potential biomarkers listed in Appendix A that biosimilar developers have used to claim biosimilarity; the reason for this lack of innovation comes from the absence of recognition of these biomarkers by the FDA.

## 11. Finding Biomarkers for Biosimilars

Understanding their PD markers is a significant aspect of developing and validating antibodies [261]. These markers, representing a drug’s bioactivity and physiological effects, are critical for guiding dosage regimens, monitoring efficacy, and predicting adverse responses [262]. However, these biomarkers have not yet been used to establish biosimilarity.

The approval of biosimilars predominantly relies on a restricted set of evidence about pharmacokinetic (PK) and pharmacodynamic (PD) similarities. Biomarkers in pharmacodynamic (PD) studies, which accurately reflect the underlying mechanisms of action of biological products, hold promise in serving as more sensitive endpoints for detecting clinically significant differences between two such products. This presents potential avenues for biomarkers previously employed as secondary and exploratory endpoints to assume crucial functions in biosimilar development initiatives. Additionally, creative approaches can be employed to develop new biomarkers for Parkinson’s disease (PD) in cases where information regarding a relevant PD biomarker is unavailable [263].

Pharmacodynamic biomarkers play an essential role in drug development as they can demonstrate the biological response of a drug on a target. For protein drugs, several biomarkers can help understand the drug’s mechanism of action, efficacy, and, sometimes, safety profile. The selection of appropriate pharmacodynamic biomarkers is essential for accurately evaluating a drug’s mechanism of action and therapeutic efficacy. Moreover, combining several biomarkers might offer a more comprehensive understanding of the drug’s effects, especially in multifactorial conditions. Harnessing these biomarkers for therapeutic and diagnostic applications requires robust experimental design, reliable analytical techniques, and a thorough understanding of the studied disease or condition. As the understanding of cellular processes deepens, the catalog of potential biomarkers will continue to expand, offering increased precision in drug development and therapeutic monitoring.

Incorporating these biomarkers effectively requires a multidisciplinary approach, where clinicians, molecular biologists, pharmacologists, and statisticians work together to evaluate the pharmacodynamic impacts of a given protein drug. Additionally, with advances in technology and an increased understanding of biology at the molecular level, newer biomarkers are continually being identified and validated.

It is essential to note that the appropriateness of a biomarker is highly dependent on the specific drug and its mechanism of action. Always, thorough validation of a biomarker for a specific context of use is critical to ensure its utility in drug development and clinical application.

The selection and validation of a pharmacodynamic biomarker will vary based on the specific protein drug and its intended therapeutic indication. The above list provides a diverse range of potential biomarkers. Still, the key is always to match the biomarker’s sensitivity and specificity with the drug’s mechanism of action and the intended clinical application. A biomarker that works well for one protein drug in a specific disease state might not be suitable for another, even if they target the same pathway. Hence, rigorous validation is essential for each specific scenario.

A broad approach to biomarker discovery, validation, and implementation is crucial. Technological advancements, particularly in omics fields (genomics, proteomics, metabolomics), enable a more comprehensive evaluation of drug impacts. Integration of these technologies and data types, often termed “systems pharmacology,” holds promise for a deeper understanding of drug action and individualized therapeutic interventions.

A practical example of a functional pharmacodynamic marker is the phosphorylation of a protein in a signaling pathway targeted by a kinase inhibitor drug. If the drug’s intended action is to inhibit a kinase, then decreased phosphorylation of its substrates after treatment would be a functional PD marker, showing that the drug is effectively inhibiting its target kinase.

It is essential to differentiate between a functional pharmacodynamic marker and other types of biomarkers. Not all biomarkers are functional in the sense of directly reflecting drug action. Some might be prognostic (indicating the likely course of a disease) or predictive (indicating the likelihood of responding to a specific treatment) but do not necessarily show the drug’s effect on the body.

New biological drug development involves extensive studies of the mode of action, the identity and function of PD biomarkers, and the factors that can alter the dose–response relationship. Table 12 lists the types of biological drugs licensed by the FDA.

More specifically, the modes of action and prospective pharmacodynamic markers identified for all FDA-licensed drugs are compiled and reported for the first time in the literature. These data are reported in Appendix A.

## 12. Conclusions

The FDA is actively advancing science-based approaches, leading to significant modifications in the approval process for biosimilars. These changes include streamlining analytical assessments, eliminating animal toxicology testing, dispensing with immunogenicity testing where it has no impact on disposition kinetics, and waiving patient testing when pharmacodynamic (PD) markers are present. As a result, the largest category of potential biosimilars, primarily monoclonal antibodies (mAbs), may still require extensive patient testing, as mandated by the BPCIA.

While novel ‘omics’ methodologies offer considerable value in developing new products, their utility for establishing biosimilarity is limited. These approaches often necessitate exhaustive research, potentially making them more cumbersome than traditional efficacy testing in patients. Nearly all biological drugs undergo investigations into their mechanisms of action when developed as new entities. In most cases, these findings can serve as potential PD biomarkers. This paper enumerates these potential PD biomarkers for all FDA-approved therapeutic proteins.

However, the FDA should select these PD biomarkers as appropriate for establishing biosimilarity rather than leaving the choice to individual developers. Such regulatory oversight would ensure a standardized and consistent assessment of biosimilar efficacy. Receptor binding, often considered a precursor to the PD response, provides a more sensitive, objective, and readily available method for evaluating the similarity between a biosimilar candidate and its reference product. Moreover, the pharmacokinetic (PK) profile often serves as a more reliable “PD biomarker” than any other identified PD markers since all PD markers are initiated in response to the disposition profile.

It is anticipated that the FDA will take decisive action—initially declaring that efficacy testing in patients is unnecessary and subsequently recommending specific PD markers, if available, for comparison in PK/PD studies. These steps would serve as the final criteria for establishing biosimilarity. This shift in scientific perspective would eliminate barriers to approving numerous biosimilars without compromising patient safety. With approximately 200 molecules identified in this paper, poised to enter the market as biosimilars, such progress would remain unattainable unless the FDA implements the suggested changes, which it has the authority to do.

## Figures and Tables

**Table 1:** Biomarker functions for biological drugs.

Function	Usage	Example
Demonstrate drug activity	To provide early evidence of a drug’s effect before overt clinical outcomes manifest.	In treating chronic myeloid leukemia (CML), the BCR-ABL tyrosine kinase inhibitor imatinib is used. The decline in BCR-ABL transcript levels in patients’ blood is a functional PD marker of the drug’s activity on its target [31].
Guide dosing	To ensure optimal drug dosing using the dose–response relationship.	For cholesterol-lowering drugs like statins, the low-density lipoprotein cholesterol (LDL-C) levels in the blood serve as a PD marker to guide dosing and assess efficacy [32].
Select patients	To identify patients likely to benefit from a specific treatment.	In some breast cancers, overexpression of the HER2 protein is observed. HER2 status serves as a functional PD marker to select patients who might benefit from trastuzumab, which targets HER2 [33].
Monitor resistance	To track the development of resistance to treatments.	In HIV treatment, the emergence of specific viral mutations can serve as PD markers indicating resistance to specific antiretroviral drugs [34].
Determine the drug mechanism of action	To confirm action through its intended mechanism.	In Alzheimer’s disease, the buildup of beta-amyloid plaques is considered a hallmark. Drugs designed to reduce beta-amyloid levels in the brain might use CSF (cerebrospinal fluid) levels of beta-amyloid as a PD marker to show the drug’s effect [35].
Validate target engagement	To demonstrate that a drug is successfully engaging with and modulating its target.	For multiple sclerosis drugs like fingolimod, a PD marker such as the number of circulating lymphocytes can indicate the drug’s effect on immune cell egress from lymph nodes [36].
Evaluate drug-induced toxicity	To monitor potential adverse effects of a drug.	In chemotherapy, monitoring the levels of liver enzymes like AST and ALT in the blood can serve as PD markers for drug-induced liver damage [37].
Optimize therapeutic window	To establish the range between the minimum effective dose and the onset of adverse effects.	For anticoagulant drugs like warfarin, the INR (International Normalized Ratio) serves as a PD marker to ensure the drug’s effect is within a therapeutic range, minimizing the risk of bleeding and clot formation [38].
Predict long-term drug effects	To predict longer-term therapeutic or adverse effects using early changes in PD markers.	In osteoporosis treatments, reducing bone resorption markers like CTX (C-terminal telopeptide) can predict longer-term benefits in bone mineral density and fracture risk [39].
Assess immune response	For immunotherapies, to gauge the body’s immune response to the treatment.	In cancer immunotherapy, the presence and proliferation of tumor-infiltrating lymphocytes (TILs) in the tumor microenvironment can serve as a PD marker to indicate the activation and targeting of the immune system against tumor cells [40].
Indicate drug combination efficacy	In combination therapies, to show the synergistic or additive effects of the combined drugs.	In treatments for tuberculosis, monitoring bacterial load in sputum samples can serve as a PD marker for the combined efficacy of multiple antimicrobial agents [41].
Track reversal of disease progression	To indicate whether a drug is not just halting but reversing disease progression.	In fibrotic diseases like idiopathic pulmonary fibrosis, measuring levels of collagen-derived peptides in blood or bronchoalveolar lavage fluid can act as PD markers, indicating the repair or degradation of fibrotic tissue [42].
Evaluate neural activity and plasticity	To track neural activity or connection changes in neurologic disorders and treatments.	For treatments aimed at Alzheimer’s or other neurodegenerative conditions, the levels of synaptic proteins or neuronal activity markers in CSF can indicate neural activity and synaptic plasticity [43].
Monitoring metabolic responses	To help track changes in metabolic pathways.	In diabetes management, measuring C-peptide levels alongside insulin can give insights into endogenous insulin production and pancreatic function [44].
Monitoring cellular senescence and aging	In treatments aiming to affect aging processes or cellular health, to track cellular senescence.	Measured levels of senescence-associated beta-galactosidase or p16^INK4a expression can act as PD markers for cellular aging or the efficacy of anti-aging treatments [45].
Evaluating epigenetic changes	To track changes in DNA methylation, histone modification, or other epigenetic markers.	In oncology, when treating with drugs targeting DNA methyltransferases, the global or gene-specific changes in DNA methylation levels can serve as PD markers [46].
Assessing drug-induced autophagy	For therapies inducing autophagy as a mechanism, to monitor the process.	When monitoring LC3B lipidation, a critical step in autophagosome formation can serve as a PD marker for autophagy activation [47].
Monitoring immune checkpoint inhibition	In cancer immunotherapy, target immune checkpoints to gauge the effectiveness of checkpoint inhibition.	In patients receiving PD-1 or PD-L1 inhibitors, monitored circulating tumor DNA (ctDNA) levels can serve as a PD marker to indicate response to therapy [48].

**Table 2:** Description of omics technologies and related analytical methods.

Analytical Method	Description
Genomics	
DNA sequencing	Determines the order of nucleotides in DNA [49].
Microarray analysis	Measures gene expression using hybridization to microarrays [50].
Whole genome sequencing	Sequences the entire genome of an organism [51].
Comparative genomics	Compares genomes of different species to identify similarities and differences [52].
Transcriptomics	
RNA-Seq	Sequences and quantifies RNA transcripts to study gene expression [53].
Microarray analysis	Measures gene expression using hybridization to microarrays [54].
Single-cell RNA-Seq	Analyzes gene expression at the single-cell level for cell heterogeneity [55].
Isoform sequencing	Focuses on the identification and quantification of alternative RNA-splicing events [56].
Proteomics	
Mass spectrometry	Identifies and quantifies proteins based on their mass-to-charge ratio [57].
2D gel electrophoresis	Separates proteins based on charge and size, allowing protein profiling [58].
Liquid chromatography (LC)	Separates proteins before mass spectrometry analysis [59].
Protein microarrays	Allows high-throughput screening of protein interactions and activities [60].
Metabolomics	
Nuclear magnetic resonance (NMR)	Measures metabolite concentrations and elucidates chemical structures [61].
Gas chromatography–mass spectrometry (GC-MS)	Separates and quantifies metabolites in the gas phase before mass spectrometry [62].
Liquid chromatography–mass spectrometry (LC-MS)	Separates and quantifies metabolites in the liquid phase before mass spectrometry [63].
Targeted metabolomics	Focuses on specific metabolites of interest for quantification [64].
Epigenomics	
DNA methylation analysis	Studies DNA methylation patterns to understand epigenetic regulation [65].
ChIP-Seq	Maps protein-DNA interactions, such as histone modifications [66].
Bisulfite sequencing	Analyzes DNA methylation status by treating DNA with bisulfite [67].
Lipidomics	
Mass spectrometry	Identifies and quantifies lipids, elucidating lipid profiles in biological samples [68].
Liquid chromatography (LC)	Separates lipids before mass spectrometry analysis [69]
Thin-layer chromatography (TLC)	Separates and identifies lipids based on their mobility on a thin layer [70].

**Table 3:** List of examples of analytical technologies broadly used.

Technology	Application
Affinity chromatography	Purification and target binding analysis.
Biacore (SPR-based technology)	Label-free interaction analysis [72].
Bottom-up MS	Mass spectrometric analysis for primary sequence analysis, evaluation of N/O-glycosylation sites, and quantification of methionine oxidation [73].
Capillary electrophoresis (CE)	Biosimilar comparability studies [74]. High-resolution separation of glycans [75].
Capillary electrophoresis–mass spectrometry	Quality control and stability of recombinant proteins in biopharmaceuticals [76,77].
Chromatography coupled with multi-angle light scattering	Absolute molar mass, size, and conformation [78].
Chemical cross-linking coupled with MS	Studying spatial arrangement and interactions within protein complexes [79].
Circular dichroism	In vivo and in vitro stability analyses [79].
Differential scanning calorimetry	Thermal stability analysis [80].
DLS	Size distribution analysis of glycoproteins [81].
Dynamic light scattering (DLS)	Size and stability analysis [82]
Electron microscopy	Visualizing glycan structures and localization [83]
Enzyme-linked immunosorbent assay (ELISA)	Detection of specific glycan–protein interactions [84]. Specific protein quantification and immunogenicity studies [85].
Enzyme-linked lectin assay	Quantitative glycan analysis [86].
Exoglycosidase sequencing	Structural characterization of glycans [87].
Flow cytometry	Cell line development and monitoring of protein expression [88].
Fluorescence spectroscopy	Sensitivity enhancement in glycan analysis [89]. Folding and conformational analysis [90].
Fourier transform infrared spectroscopy (FTIR)	Secondary structure analysis and stability monitoring [91,92]. Designing more effective protein-based therapies through metabolite profiling [82]. Analysis of glycan structure [93].
Gas chromatography–mass spectrometry	Analysis of volatile derivatives of glycans [94]. Understanding host-pathogen interactions for recombinant vaccine development [95,96]
Gel electrophoresis, 2D	Separation and identification of proteins [97,98].
Glycan microarrays	High-throughput analysis of protein–glycan interactions [99].
Glycan sequencing using MS/MS	Sequential identification [100].
Glycoproteomic analysis	Integrative approach for comprehensive glycoprotein study [101].
High-performance liquid chromatography (HPLC)	Separation of glycan structures [102].
Hydrogen–deuterium exchange mass spectrometry	Conformational dynamics and higher-order structure analysis [103].
Hydrophilic interaction liquid chromatography (HIC)	Separation of polar glycans [88]. Analysis of hydrophobicity and aggregation [104].
IEF	Separation of glycoproteins by isoelectric point [105].
Immunoassays	Pharmacokinetic and pharmacodynamic studies [106].
Immunohistochemistry	Localization of specific glycans in tissues [107].
Immunoprecipitation and pull-down assays	Protein interaction studies [108].
Ion exchange chromatography	Charge heterogeneity analysis [109].
Isoelectric focusing (IEF)	Protein separation based on isoelectric point [110].
Lectin affinity chromatography	Separation of glycans using specific binding proteins [111].
Liquid chromatography–mass spectrometry	Comprehensive protein characterization [112]. Improving industrial protein production yields Environmental stress response in recombinant protein production [113].
Mass spectrometry (MS)	Characterization and post-translational modification analysis [114]. Structural analysis of glycan [115].
Mass spectrometry and NMR	The optimization of expression systems for protein production [116].
Mass spectrometry selected reaction monitoring	Targeted protein quantification in biosimilar development [117].
Mass spectrometry, tandem	Sequential fragmentation for glycan structure elucidation [118].
Mass spectrometry, ultra-performance liquid chromatography	Assessing the impact of protein therapeutics on metabolic pathways [119].
Matrix-assisted laser desorption/ionization (MALDI-MS)	Mass determination of glycan [120]. Rapid identification and characterization of proteins [121].
Multi-angle light scattering (MALS)	Molar mass and size distribution [122]. Characterizing size and composition [123].
Multi-angle light scattering (MALS)	Molar mass and size distribution [122].
N-Terminal sequencing	Analysis of protein sequence and modifications [124].
Mass spectrometry, native	Structural characterization and complex formation analysis [125].
NMR spectroscopy	Detailed structural analysis of glycan [126]. Conformational dynamics and structural analysis [127]. Investigating protein–metabolite interactions [128].
Optical glycan biosensors	Real-time monitoring of glycan–protein interactions [129].
Peptide mapping and fingerprinting	Identification and characterization of proteins [130].
Protein microarrays	High-throughput analysis of protein functions and interactions [131].
Reverse-phase liquid chromatography	Separation of glycopeptides and glycoproteins [132]. Analysis of protein purity and heterogeneity [133].
Size-exclusion chromatography	Protein aggregation and purity assessment [134].
SPR	Real-time glycan–protein interaction analysis [135].
Stable isotope labeling	Quantitative glycomics [136]. Quantitative proteomics for expression analysis [137].
Surface plasmon resonance (SPR)	Protein interaction studies [138].
X-ray crystallography and nuclear magnetic resonance (NMR) spectroscopy	3D structure determination of glycoproteins [139]. Structural analysis and 3D modeling [140].
Yeast two-hybrid system	Protein–protein interaction mapping [141].

**Table 4:** Examples of applications of proteomic biomarkers.

Examples of Use of Proteomics Biomarkers	
Antibody-drug conjugates (ADCs)	Brentuximab vedotin, an ADC used for Hodgkin’s lymphoma and systemic anaplastic large cell lymphoma, delivers the cytotoxic drug monomethyl auristatin E (MMAE) to CD30-expressing cells, and the measurement of MMAE can serve as a marker of target engagement [143].
Antigen-antibody complex	The formation of antigen–antibody complexes provides direct evidence of target engagement. For example, in the case of adalimumab, an anti-tumor necrosis factor (TNF)-α antibody, the serum levels of the adalimumab-TNFα complex can be measured as evidence of the drug binding to its target [144].
Antigenic modulation	This refers to the downregulation or loss of antigen expression on the cell surface in response to antibody binding and can be used as a marker of monoclonal antibody (mAb) engagement. Rituximab, a monoclonal antibody against the CD20 antigen on B cells, causes antigenic modulation, decreasing CD20 expression and indicating rituximab engagement [145].
Binding of mAbs to Fc receptors	The Fc region of mAbs can bind to Fc receptors on immune cells. This binding can modulate the activity of these cells, making Fc-receptor occupancy a valuable PD marker. The occupancy of RIIIa on natural killer cells by rituximab can be used as a PD marker [146].
Cell proliferation markers	mAbs may also be designed to inhibit cell proliferation. Here, decreased cell proliferation markers, such as Ki-67, can indicate successful target engagement [147].
Circulating tumor antigen levels	In cancer therapy, mAbs are often designed for binding to specific tumor antigens. A reduction in the levels of these circulating antigens following mAb therapy can serve as a marker of target engagement. For instance, CA-125 levels in patients with ovarian cancer have been treated with mAbs targeting the CA-125 antigen [148].
Complement system alterations	mAbs can modulate the complement system. Eculizumab, a mAb that inhibits complement component C5, reduces hemolytic activity and can be used as a PD marker [149].
Cytokine release syndrome	mAbs, particularly those targeting immune cells, increase the release of specific cytokines. For instance, administration of the anti-CD28 mAb TGN1412 releases many cytokines, such as interleukin (IL)-2 and interferon (IFN)-γ, which could be monitored as PD markers. Measuring cytokines, such as IL-2, IL-6, or TNF-α, can estimate target engagement. This is particularly relevant for immunomodulatory mAbs such as ipilimumab, which can increase circulating cytokine levels upon engagement with its target, cytotoxic T lymphocyte-associated (CTLA-4) [150].
Fluorescent tag	Flow cytometry can be a valuable tool for assessing target engagement when the target of a mAb is expressed on cell surfaces. Labeling the mAb with a fluorescent tag confirms its binding to the target cells in a sample. This has been utilized in therapies, such as those using rituximab, wherein binding to CD20+ B cells can be confirmed using flow cytometry [151].
Gut microbiota alterations	Specific mAb therapies can alter gut microbiota, serving as functional response markers. Vedolizumab, a mAb against the α4β7 integrin used in treating inflammatory bowel disease, can restore gut microbial diversity, indicating a functional response to therapy [152].
Immune response markers	Some mAbs stimulate immune responses against specific antigens. Hence, increased antibodies against the target antigen in the patient’s serum can serve as a target engagement marker. For instance, palivizumab, a mAb that prevents respiratory syncytial virus (RSV) infection in high-risk infants, engages its target through anti-RSV antibodies in the patient’s serum [153]. Immune response can be measured as a functional response marker. For instance, ipilimumab, a mAb that targets the immune checkpoint protein CTLA-4, is the most widely used mAb.

**Table 5:** Methods of identifying glycans and their applications.

Application	Example
Bioanalytical and bioinformatics	Data integration at different structural levels to identify various glycoforms of recombinant human chorionic gonadotropin (r-hCG), urinary hCG (u-hCG), and recombinant follicle stimulating hormone (r-hFSH) revealed that these biopharmaceuticals differ considerably in their glycosylation patterns [156].
Resolution discrepancies	Between high-resolution native and glycopeptide-centric mass spectrometric approaches for the glycosylation of erythropoietin variants [157].
Glycan microheterogeneity	To identify multiple glycosylation sites in the vascular endothelial growth factor IgG (VEGFR-IgG) fusion protein to understand the functional significance of each glycosylation pattern [158].
Glycoforms	Several glycoforms using hybrid high-performance liquid chromatography–MS approaches [159], such as 24 glycoengineered erythropoietin variants with varying glycan branching and sialylation levels, are crucial parameters for biotherapeutic efficacy.
NMR	Identification of glucose-induced glycation in mAbs and other proteins using NMR spectroscopy. [160].
Novel glycoforms	Identification of novel glycosylations in human-serum-derived factor IX. [161].
Mass spectral profiling	The N-linked, O-linked, ganglioside, and glycosaminoglycan compound classes and the tandem mass spectrometry of glycans have led to spectral glycoproteomics [162].
N-glycosylation profile	Analysis of trastuzumab biosimilar candidates using normal-phase liquid chromatography and matrix-assisted laser desorption/ionization time-of-flight MS [163].
Microheterogeneity	Composite glycosylation profiles and other microheterogeneities in intact mAbs via high-resolution native MS using a modified Orbitrap [164].
Targeted site-specific quantitation	N-and O-glycopeptides using 18O-labeling and product ion-based MS [165].
Hybrid MS	Approaches in glycoprotein analysis and their usage in scoring biosimilarity [166].

**Table 6:** Applications of transcriptomics.

Application	Example
Gene expression profiling	Simultaneous measurement of the expression levels of several genes to create a global picture of cellular functions.
Optimization of host cells for protein expression	Optimize host cells to improve recombinant protein expression [170].
Identifying suitable expression systems	Selection of optimal expression system [171].
Understanding protein function and interactions	How proteins function and interact within cellular networks [172].
Monitoring quality control in biopharmaceutical production	Manufacturing QC [173].
Improving protein solubility and folding	Enhance the solubility and folding of recombinants [174].
Tailoring protein post-translational modifications (PTMs)	To produce proteins with the desired PTMs [175].
Enhanced yield in industrial protein production	To enhance the yield of recombinant proteins for industrial applications [176].
Studying protein stability and degradation	Design recombinant proteins with enhanced stability [177].
Personalized medicine and therapeutics	Patient-specific proteins based on individual gene expression profiles [178].

**Table 7:** Examples of genomics applications in recombinant therapeutic protein development.

Application	Example
Improving expression systems	Researchers can optimize expression systems for recombinant protein production by studying host cell genomes [183].
Designing recombinant proteins	Genomics can be used to identify and design recombinant proteins with the desired functions [184].
Enhancing protein stability	Understanding the genomic context can aid in designing recombinant proteins with enhanced stability and activity [185].
Personalized medicine	Personalized genomics allows the development of recombinant proteins tailored to the genetic profiles of individual patients [186].
Metabolic engineering	Genomic insights can guide the redesign of metabolic pathways to efficiently produce recombinant proteins in microbial systems [187].
Biomarker discovery	Genomics aids in the identification of biomarkers that can be targeted with recombinant proteins for diagnostic or therapeutic purposes [188].
Understanding protein function	Comparative genomics can elucidate the functions of proteins by identifying conserved sequences and structures [189].
Enhanced protein folding	Genomic data can be used to understand and improve the folding of recombinant proteins [190].
Development of novel therapeutics	Genomics is used to identify potential targets of therapeutic recombinant proteins [191].
Cell line optimization	Cell line optimization results from: (i) research applied to parental, non-recombinant cell lines; (ii) systems-level datasets generated with recombinant cell lines; (iii) datasets linking phenotypic traits to relevant biomarkers; (iv) data depositories and bioinformatics tools; and (v) in silico model development [192].

**Table 8:** Applications of epigenomics in the development of therapeutic proteins.

Application	Example
Optimization of expression systems	Understanding and manipulating epigenetic markers can enhance recombinant proteins’ expression in host cells [196].
Production of recombinant proteins with specific modifications	Epigenomic control enables the production of recombinant proteins with functionally essential PTMs [197].
Development of recombinant proteins for epigenetically targeted therapies	Epigenomics guides the discovery and development of recombinant proteins that target specific epigenetic modifications involved in various diseases [198].
Studying the epigenetic control of protein function	Recombinant proteins can be used to study how epigenetic modifications regulate endogenous proteins, providing insights into their functions and control [199].
Recombinant epigenetic modifiers for research	Producing recombinant proteins involved in epigenetic modifications, such as methyltransferases, may be helpful for research and drug development [200].
Modeling diseases	Using epigenomic information for modifying host cells to produce recombinant proteins allows the creation of more accurate disease models, particularly for conditions in which epigenetic alterations play a crucial role [201].
Quality control and stability of biopharmaceuticals	Epigenomic control may improve the quality and stability of recombinant proteins for biopharmaceutical applications [75].

**Table 9:** Examples of metabolomics applications.

Application	Example
Immune response against tumor cells in patients with melanoma	Increases in absolute lymphocyte counts are observed in response to therapy and can be a marker of functional immune response [205].
Lab values alteration	Changes in specific laboratory values can serve as functional response markers for certain conditions. For example, in patients with rheumatoid arthritis treated with the anti-TNFα mAb, adalimumab, reductions in serum C-reactive protein levels and erythrocyte sedimentation rate, which are markers of inflammation, indicate a positive response to therapy [206].
Modulation of T-cell response	Some mAbs, particularly immune checkpoint inhibitors such as pembrolizumab, function by modulating the T-cell response, which can be measured using T-cell activation markers, such as CD137, or by enumerating antigen-specific T-cells [207].
Alteration in serum immunoglobulin levels	Some mAbs can cause changes in the serum levels of immunoglobulins, which can serve as PD markers. Rituximab, an anti-CD20 mAb, decreases serum immunoglobulin levels, which can be monitored clinically [208].
PK and PD correlation	The correlation between PK properties, such as serum drug concentration and PD markers, is a significant component of mAb engagement. This elucidates the dose–response relationship for adjusting the dosing regimens. For instance, with infliximab, an anti-TNFα mAb used in treating autoimmune diseases, measuring the serum drug concentration and correlating this with the clinical response and anti-TNFα activity can indicate effective drug-target engagement [209].

**Table 10:** Role of omics technologies and their rationale in the development of biosimilars.

Omics Technology	Role	Rationale
Proteomics	Determining the protein expression profile, post-translational modifications (like glycosylation), and protein–protein interactions of the biosimilar compared to the reference product.	Minor differences in protein structure or modifications can impact the efficacy.
Transcriptomics	Analyzing the gene expression profile of cells producing the biosimilar ensures that the cellular machinery has the therapeutic protein in a manner consistent with the reference product.	Differences in gene expression may hint at differences in protein product production, folding, or modification.
Metabolomics	Examining the metabolic profile of the biosimilar-producing cells.	The metabolic state of a cell can influence the final product’s quality and consistency. For instance, changes in nutrient levels can impact the glycosylation patterns of proteins.
Genomics	Ensuring the genetic stability of the cell line producing the biosimilar.	Over time, cell lines might undergo genetic drift, which can impact the product’s quality, consistency, and efficacy.
Microbiomics	Understanding the microbiome can be essential if the biological product has a microbial origin (like some recombinant proteins produced in bacteria).	Microbial contaminants or shifts in the microbial population can influence the final product’s quality and safety.
Phosphoproteomics	Analyzing phosphorylation patterns on proteins can be critical for some biologics’ function or stability.	Changes in phosphorylation can affect protein activity, stability, or interaction with other proteins.

**Table 11:** Binding receptors for pharmacodynamic markers.

mAb (Brand)	Receptor
Abciximab (ReoPro) [219]	GPIIb/IIIa
Adalimumab (Humira) [220]	TNFα
Alemtuzumab (Lemtrada) [221]	CD52
Atezolizumab (Tecentriq) [222]	PD-L1
Basiliximab (Simulect) [223]	CD25
Belimumab (Benlysta) [224]	BLyS
Bevacizumab (Avastin) [225]	VEGF
Cetuximab (Erbitux) [226]	EGFR
Daclizumab (Zinbryta) [227]	CD25
Daratumumab (Darzalex) [228]	CD38
Denosumab (Prolia) [229]	RANKL
Dupilumab (Dupixent) [230]	IL-4Rα
Eculizumab (Soliris) [149]	C5
Infliximab (Remicade) [231]	TNFα
Ipilimumab (Yervoy) [232]	CTLA-4
Nivolumab (Opdivo) [233]	PD-1
Obinutuzumab (Gazyva) [234]	CD20
Ofatumumab (Arzerra) [235]	CD20
Omalizumab (Xolair) [236]	IgE
Palivizumab (Synagis) [237]	RSV F protein
Pembrolizumab (Keytruda) [238]	PD-1
Rituximab (Rituxan) [239]	CD20
Sarilumab (Kevzara) [240]	IL-6R
Secukinumab (Cosentyx) [241]	IL-17A
Tocilizumab (Actemra) [242]	IL-6R
Trastuzumab (Herceptin) [33]	HER2/neu
Vedolizumab (Entyvio) [243]	α4β7 integrin

**Table 12:** Current licensed biological drugs by the FDA (https://drugs.ncats.io/ (accessed on 12 August 2023).

Type	Count
Fab	1
Toxin	1
Carrier protein	1
Single-domain antibody	1
Fusion proteins	1
Bispecific antibody	3
Coagulation factor	4
Cytokine	4
Peptide	4
Growth factor	4
Enzyme	9
Enzyme inhibitor	11
Hormone	11
Monoclonal antibody conjugate	13
Monoclonal antibody	96
Total	164

## Data Availability

Data is contained within the article and Appendix A.

## Appendix A

**Table A1:** Mode of action of therapeutic proteins.

Mode of Action	Biomarker Potential
AMPK and mTORC1 Signaling	Monitoring these central energy sensors and regulators can be vital for drugs targeting cellular energy status or metabolic health. [264]
Angiogenesis Indicators	If a protein drug affects blood vessel formation, angiogenic factors like VEGF can be used as biomarker. [265]
Apoptosis Markers	Evaluation of cell death can be instrumental for drugs designed to induce or inhibit apoptosis. Markers such as caspase activation or phosphatidylserine externalization can be employed. [266]
Autophagy Markers	LC3-II and p62/SQSTM1, for drugs modulating autophagic activity. [267]
Autophagy-lysosomal Pathway Markers	Monitoring markers like p62/SQSTM1 or LAMP1 can give insights into the autophagy-lysosomal activity upon drug treatment. [268]
Blood Coagulation Factors	For protein drugs affecting hemostasis, measurement of specific clotting factors or clotting times might be used. [269]
Bone Turnover Markers	For protein drugs acting on the skeletal system, bone resorption or formation markers can provide insight into their effect. [270]
Calcium Signaling	Monitoring intracellular calcium flux and associated proteins can be important for drugs that modulate calcium homeostasis or signaling pathways. [271]
Cell Cycle Regulators	Drugs aiming at modulating the cell cycle might alter levels or activities of cyclins, cyclin-dependent kinases, or associated inhibitors. [272]
Cell Metabolism	Assessing the metabolic profile of cells or tissues after drug treatment, for instance, glucose uptake, lactate production, or ATP level. [273]
Cell Surface Markers	These markers can be evaluated for drugs targeting cell surface proteins or for those that induce phenotypic changes in cells. [274]
Cellular Apoptosis or Proliferation	Some protein drugs may induce or inhibit apoptosis or cell proliferation, which can be quantified. [275]
Cellular Signaling Pathways	Assessment of downstream or upstream signaling pathways that might be affected by the protein drug. MAPK, PI3K/AKT, or JAK/STAT pathways. [276]
Changes in specific cell populations	Especially in immunology, a protein drug can lead to the proliferation or reduction of specific cell populations. [277]
Circadian Rhythms	For protein drugs affecting cellular or physiological rhythms, markers related to circadian clock genes such as PER, CRY, or CLOCK might be relevant. [278]
Complement Activation	For specific therapeutic proteins, activation or inhibition of the complement system can serve as a pharmacodynamic readout. [279]
Cytokine Levels	Many protein drugs target specific cytokines or have effects on cytokine levels. [280]
DNA Damage and Repair Markers	γH2AX and other proteins associated with DNA damage response can be relevant for drugs targeting genomic stability. [281]
Drug Concentration	Although this is more of a pharmacokinetic parameter, the concentration of the drug in the bloodstream can sometimes serve as a surrogate for its pharmacodynamic effects, significantly when the concentration closely correlates with the drug’s effect. [282]
Endocannabinoid System Markers	Components include CB1 and CB2 receptors or endocannabinoids (anandamide, 2-AG) for drugs affecting this system. [283]
Endocrine Biomarkers	For protein drugs affecting the endocrine system, hormones or hormone precursors might be potential pharmacodynamic indicators. [284]
Endocytosis and Exocytosis Metrics	Protein drugs targeting cell trafficking mechanisms might alter the rates of endocytosis or exocytosis, which can be tracked using various cellular assays. [285]
Endogenous Antioxidant Enzymes	Superoxide dismutase (SOD), catalase, and glutathione peroxidase levels can be tracked for oxidative stress modulation. [286]
Endoplasmic Reticulum (ER) Stress Markers	GRP78/BiP, CHOP, XBP1, ATF6 for drugs influencing ER homeostasis or targeting diseases related to protein misfolding. [287]
Endosome Trafficking	Protein drugs that interfere with endosomal pathways can be monitored for their effects using markers of early, late, and recycling endosomes. [288]
Endothelial Activation Markers	For drugs impacting vascular inflammation or barrier function, such as E-selectin, ICAM-1, and VCAM-1. [289]
Enzyme Activity	If the protein drug targets an enzyme, measuring the change in enzyme activity can be an effective biomarker. [290]
Epigenetic Markers	Changes in DNA methylation, histone modification, or other epigenetic markers might indicate a response to certain protein drugs. [290]
Exosome Release and Composition	Certain protein drugs can influence exosomes and their cargo (RNA, protein, lipids), especially those impacting intercellular communication. [291]
Extracellular Matrix (ECM) Components	Matrix metalloproteinases (MMPs) and tissue inhibitors of metalloproteinases (TIMPs) are relevant for tissue remodeling or cancer invasion. [292]
Fatty Acid Oxidation (FAO) Rates	Drugs targeting metabolic states might shift cells between carbohydrate and fatty acid metabolism. [293]
Flow Cytometry	This is particularly relevant for drugs that target cells of the immune system. Flow cytometry can provide insights into cell numbers, phenotypes, and functions. [273]
Functional Assays	Depending on the intended drug action, functional assays can be developed. For instance, if a protein drug aims to inhibit a specific cellular function, assays can be set up to measure that specific function. [155]
Gene Expression Profiles	Transcriptomics can reveal the downstream effects of a protein drug on cellular gene expression. [294]
Glycolytic versus Oxidative Metabolism	Assessing the switch between glycolytic and oxidative metabolism can be crucial for drugs targeting metabolic diseases or cancer. [295]
Glycosylation Patterns	Alterations in the glycosylation patterns of cells or proteins can directly or indirectly affect some protein drugs. [296]
Gut Microbiota Composition	Sequencing or metabolomic profiles of gut bacteria can be helpful for drugs impacting the gut environment. [297]
Heat Shock Proteins (HSPs)	As molecular chaperones, changes in HSP levels can indicate cellular stress responses or protein homeostasis disruptions. [298]
Heat Shock Proteins (HSPs)	These proteins respond to cellular stress and can be targets or indicators for several drugs, especially in protein-misfolding diseases. [298]
Histone Modifications	Epigenetic changes, like histone acetylation or methylation, can be markers for drugs targeting chromatin remodeling or gene expression. [299]
Hormone Levels	Assessment of specific hormone levels, like insulin, glucagon, or thyroid hormones, can indicate drug impact on endocrine systems. [300]
Hypoxia Indicators	For protein drugs affecting cellular responses to oxygen deprivation, markers like HIF-1α can be interesting. [301]
Imaging Biomarkers	Techniques like MRI, PET, and CT can be used to measure the effects of protein drugs at the tissue or organ level. [302]
Immune Response Markers	The immune system might mount an antibody response for protein drugs, especially those foreign to the human body. Monitoring anti-drug antibodies (ADAs) can be a biomarker for potential immunogenicity issues. [303]
Inflammasome Activation	Monitoring inflammasome components can be helpful in drugs targeting inflammatory conditions or diseases like Alzheimer’s. [304]
Ion Channel Activity	For protein drugs targeting ion channels, the measurement of ion flux or electrical properties of cells could directly indicate drug action. [305]
Iron Metabolism Markers	Ferritin, transferrin, and hepcidin for drugs modulating iron homeostasis. [306]
Levels of circulating drug target	If the target of the protein drug circulates in the bloodstream (like a soluble receptor or ligand), measuring its levels can serve as a biomarker. [246]
Ligand-Receptor Interactions	Investigating a protein drug’s binding dynamics and affinity to its target receptor can provide insights into its effectiveness. [307]
Lipidomic Profile	Analyzing the cellular lipid composition can be informative, especially for drugs impacting lipid metabolism or signaling. [308]
Lipophagy Markers	Indicators of lipid droplet autophagy crucial for lipid metabolism-related conditions. [309]
Lysosomal Enzymes	The levels and activity of the specific lysosomal enzymes can be essential biomarkers for enzyme replacement therapies in lysosomal storage disorders. [310]
Markers of Fibrosis	In conditions like liver or lung fibrosis, protein drugs might target fibrogenesis, and thus, markers such as tissue collagen or specific matrix proteins can serve as indicators. [311]
Metabolic Enzymes	Monitoring the levels or activities of critical metabolic enzymes, such as those involved in glycolysis or the TCA cycle, can provide insights into the metabolic state of cells upon drug treatment. [312]
MicroRNAs (miRNAs)	Changes in the expression of specific miRNAs can serve as biomarkers since they play pivotal roles in gene regulation and might be influenced by protein drugs. [313]
Mitochondrial Dynamics	Assessing mitochondrial morphology and dynamics can indicate cellular health and metabolism, especially for drugs targeting these organelles. [314]
Mitophagy Indicators	Monitoring mitophagy, a process to degrade damaged mitochondria, can be helpful in drugs targeting cellular health. [315]
mRNA Splicing Markers	Such as components of the spliceosome for drugs modulating RNA splicing or targeting splicing-related diseases. [316]
mTOR Signaling	The mechanistic target of the rapamycin (mTOR) pathway, central to cell growth and metabolism, might be affected by certain protein drugs. Monitoring components like p70S6 kinase or 4E-BP1 can be informative. [317]
Myelination Markers	Proteins like MBP or PLP can be tracked for drugs targeting neurodegenerative diseases or demyelinating conditions. [318]
NAD+/NADH Ratio	A marker for cellular redox state and metabolism, especially relevant for aging
Neural Activity Markers	c-Fos, Arc, or immediate early genes can be indicators of neural activity and synaptic plasticity. [319]
Neurotransmitter Levels	If the drug has a neurological target, neurotransmitter levels in the central nervous system or peripheral tissues can be evaluated. [320]
Neurotrophic Factors	In neurodegenerative diseases, protein drugs might aim to modulate the levels of neurotrophic factors like BDNF, NGF, or GDNF. [321]
NO (Nitric Oxide) Production	Relevant for cardiovascular or immunomodulatory drugs, NO levels can indicate endothelial function and inflammatory states. [322]
Nrf2-Keap1 Pathway	Tracking the Nrf2-Keap1 pathway components can be vital for drugs that modulate oxidative stress. [323]
Nucleotide Metabolites	Monitoring the levels of specific nucleotide metabolites can indicate cellular activity or stress in response to certain protein drugs. [324]
Oxidative Phosphorylation (OXPHOS) Metrics	OXPHOS or mitochondrial health markers can be relevant for protein drugs targeting mitochondrial function. [325]
Oxidative Stress Markers	Oxidative stress plays a role in numerous diseases, and markers like reactive oxygen species (ROS) or antioxidant levels can be used to assess drug effects. [326]
Oxysterols	Such as 24(S)-hydroxycholesterol 27-hydroxycholesterol for drugs targeting cholesterol metabolism or diseases like Niemann-Pick type C. [327]
Peroxisome Proliferators-Activated Receptors (PPARs)	As metabolic regulators, PPARs can be markers for drugs impacting lipid metabolism or inflammation. [328]
Pharmacogenomic Biomarkers	Some patients might respond differently to protein drugs based on genetic variations. Exploring these can provide insights into efficacy and safety. [329]
Phosphorylation status of proteins	The activation or deactivation of specific signaling pathways can be tracked by looking at the phosphorylation status of essential proteins. [330]
Proteasome Activity	Assessing proteasomal activity can be insightful for protein drugs that modulate protein degradation. [331]
Proteomic Analysis	To assess broader proteome for changes in protein levels or post-translational modifications upon drug treatment. [57]
Reactive Oxygen Species (ROS) Levels	As an indicator of oxidative stress, ROS can be monitored for drugs that either induce or counteract cellular stress. [332]
Receptor Occupancy	Measuring the degree to which a protein drug binds to its target receptor can be a direct biomarker of its activity. [333]
Senescence-associated Secretory Phenotype (SASP) Factors	Monitoring factors associated with cellular senescence might be relevant for drugs targeting aging or oncogenesis. [334]
Sirtuin Activity	Monitoring sirtuin proteins can be essential for drugs modulating cellular longevity or metabolic health. [335]
Telomerase Activity	Telomerase activity or telomere length might be relevant biomarkers for drugs targeting cancer or aging processes. [336]
Tight Junction Proteins	Markers like claudins, occludin, and ZO-1 are relevant for drugs targeting barrier integrity, such as in gut or blood-brain barrier conditions. [337]
Tissue Repair and Regeneration Markers	For protein drugs that facilitate tissue healing, markers of tissue repair or stem cell activation might be relevant. [338]
Tumor Microenvironment Components	Factors like TGF-beta, PD-L1, and various cytokines/chemokines for drugs targeting cancer immune evasion. [339]
Unfolded Protein Response (UPR) in the ER	For protein drugs that might induce ER stress, tracking components of the UPR can be informative. [340]
Wnt Signaling Pathway Components	Tracking this pathway can be crucial for drugs that modulate developmental processes, tissue regeneration, or certain cancers. [341]

**Table A2:** Reported PD Biomarkers of approved therapeutic proteins.

No	Protein Drug	Pharmacodynamic Marker
1.	Abatacept (Orencia)	T cell proliferation & co-stimulation [342]
2.	Adalimumab (Humira)	TNF-alpha levels & inflammatory cytokine reduction [343]
3.	Aducanumab (Aduhelm)	Beta-amyloid plaques in the brain [344]
4.	Aflibercept (Eylea)	VEGF levels, Central retinal thickness [345]
5.	Agalsidase Alfa (Replagal)	Lyso-Gb3 levels, Kidney function [346]
6.	Agalsidase Beta (Fabrazyme)	Lyso-Gb3 levels, Kidney function [347]
7.	Albiglutide (Tanzeum)	Blood glucose & GLP-1 levels [348]
8.	Albutrepenonacog Alfa (Idelvion)	Factor IX activity levels [349]
9.	Aldesleukin (Proleukin)	T cell count, IL-2 levels [350]
10.	Alefacept (Amevive)	CD4 and CD8 memory T-cell count [351]
11.	Alemtuzumab (Lemtrada)	CD52-expressing cell count [352]
12.	Alglucerase (Ceredase)	Gaucher cell count, Chitotriosidase levels [353]
13.	Alirocumab (Praluent)	LDL cholesterol levels [354]
14.	Alpha-1-proteinase inhibitor (Prolastin, etc.)	Alpha-1 antitrypsin levels, Neutrophil elastase activity [355]
15.	Alteplase (Activase)	Fibrinolytic activity, clot dissolution [356]
16.	Amivantamab (Rybrevant)	EGFR and MET signaling inhibition [357]
17.	Anakinra (Kineret)	IL-1β levels [358]
18.	Ancestim (Stemgen)	CD34+ cell count in peripheral blood [359]
19.	Andexanet Alfa (Andexxa)	Reversal of factor Xa inhibitors [360]
20.	Anifrolumab (Saphnelo)	Type I interferon gene signature [361]
21.	Anistreplase (Eminase)	Fibrinolytic activity [362]
22.	Ansuvimab (Ebanga)	Reduction in viral load of Ebola virus [363]
23.	Atezolizumab (Tecentriq)	PD-L1 expression on tumor & immune cells [364]
24.	Avelumab (Bavencio)	PD-L1 expression & T cell activation [365]
25.	Benralizumab (Fasenra)	Reduction in eosinophil counts [366]
26.	Bermekimab (Xilonix)	IL-1α levels [367]
27.	Bevacizumab (Avastin)	VEGF level & microvessel density [368]
28.	Bezlotoxumab (Zinplava)	Reduction in C. difficile infection recurrence [369]
29.	Bimekizumab (Bimzelx)	IL-17A and IL-17F levels [370]
30.	Bivalirudin (Angiomax)	Thrombin activity [371]
31.	Blinatumomab (Blincyto)	CD19+ B cell count [372]
32.	Bone Morphogenetic Proteins (BMPs)	Bone density or new bone formation [373]
33.	Botulinum Toxin Type A (Botox)	Neuromuscular transmission inhibition [374]
34.	Botulinum Toxin Type B (Myobloc)	Neuromuscular transmission inhibition [375]
35.	Brodalumab (Siliq)	IL-17 receptor A occupancy [376]
36.	Brolucizumab (Beovu)	VEGF levels, Central retinal thickness [377]
37.	Burosumab (Crysvita)	Serum phosphorus levels [378]
38.	Calaspargase pegol (Asparlas)	Asparagine levels [379]
39.	Canakinumab (Ilaris)	IL-1β levels & CRP [380]
40.	Cetuximab (Erbitux)	EGFR expression & phosphorylation [226]
41.	Chymopapain (Chymodiactin)	Disc volume reduction [381]
42.	Coagulation factor IX (BeneFIX)	Factor IX clotting activity [382]
43.	Coagulation Factor VIIa (NovoSeven)	Clotting activity [383]
44.	Collagenase (Santyl)	Degrades necrotic tissue [384]
45.	Conestat alfa (Ruconest)	Bradykinin levels [385]
46.	Corticotropin (Acthar)	Adrenal gland stimulation [386]
47.	Cosyntropin-ACTH(1-24) (Cortrosyn)	Adrenal gland stimulation [387]
48.	Crizanlizumab (Adakveo)	P-selectin inhibition [388]
49.	Darbepoetin alfa (Aranesp)	Hemoglobin or hematocrit level [389]
50.	Denosumab (Prolia, Xgeva)	RANKL inhibition & bone turnover markers [390]
51.	Denosumab (Prolia)	Bone mineral density & serum C-telopeptide [390]
52.	Dupilumab (Dupixent)	IL-4 and IL-13 signaling pathways [391]
53.	Durvalumab (Imfinzi)	PD-L1 expression in tumor cells [392]
54.	Eculizumab (Soliris)	Complement component C5 activity [393]
55.	Edrecolomab (Panorex)	EpCAM expression [394]
56.	Efalizumab (Raptiva)	CD11a expression [395]
57.	Efgartigimod alfa	IgG reduction [396]
58.	Elapegademase (Revcovi)	ADA enzyme activity [397]
59.	Elosulfase Alfa (Vimizim)	GAG reduction [398]
60.	Elotuzumab (Empliciti)	SLAMF7 expression in myeloma cells [399]
61.	Emapalumab (Gamifant)	IFNγ levels [400]
62.	Emicizumab (Hemlibra)	Factor IXa and factor X bridging [401]
63.	Enfortumab vedotin (Padcev)	Nectin-4 expression [402]
64.	Erenumab (Aimovig)	CGRP receptor binding and inhibition [403]
65.	Erythropoietin (EPO)	Hemoglobin or hematocrit level [404]
66.	Eteplirsen (Exondys 51)	Dystrophin production in muscle tissue [405]
67.	Evolocumab (Repatha)	LDL cholesterol levels [406]
68.	Fibrinolysin (Elase)	Fibrin degradation [407]
69.	Filgrastim (Neupogen)	Neutrophil count [408]
70.	Follitropin (Follistim, Gonal-f)	Follicular development, Estradiol levels [409]
71.	Fremanezumab (Ajovy)	CGRP levels [410]
72.	Galcanezumab (Emgality)	CGRP levels [411]
73.	Galcanezumab (Emgality)	CGRP binding [412]
74.	Galsulfase (Naglazyme)	Urinary glycosaminoglycan levels [413]
75.	Gemtuzumab ozogamicin (Mylotarg)	CD33 antigen expression [414]
76.	Girentuximab	CAIX expression [415]
77.	Glatiramer acetate (Copaxone)	Immune modulation; T-cell response [416]
78.	Glucagon recombinant (GlucaGen)	Blood glucose elevation [417]
79.	Glucarpidase (Voraxaze)	Methotrexate levels reduction [418]
80.	Golimumab (Simponi)	TNFα inhibition [419]
81.	Growth Hormone	IGF-1 (Insulin-like Growth Factor 1) level [420,421]
82.	Guselkumab (Tremfya)	IL-23 levels & PASI score [422]
83.	Human C1-esterase inhibitor (Berinert, Cinryze)	C1-INH levels and activity [423]
84.	Ibalizumab (Trogarzo)	HIV-1 viral load & CD4+ T-cell count [353]
85.	Imiglucerase (Cerezyme)	Glucocerebroside levels, macrophage activity [424]
86.	Inebilizumab (Uplizna)	B-cell depletion [425]
87.	Infliximab (Remicade)	TNF-alpha levels & CRP [426]
88.	Inotuzumab ozogamicin (Besponsa)	CD22 expression [427]
89.	Insulin Regular (Humulin R, etc)	Glucose levels [428]
90.	Interferons	Expression of interferon-responsive genes [429]
91.	Ipilimumab (Yervoy)	T-cell activation [430]
92.	Isatuximab (Sarclisa)	CD38 expression [431]
93.	Itolizumab (Alzumab)	CD6 expression [432]
94.	Ixekizumab (Taltz)	PASI score, serum IL-17A levels [433]
95.	Lanadelumab (Takhzyro)	Plasma kallikrein activity [434]
96.	Lanadelumab (Takhzyro)	Plasma kallikrein inhibition [435]
97.	Laronidase (Aldurazyme)	Reduction of glycosaminoglycans [436]
98.	Lepirudin (Refludan)	Inhibition of thrombin [437]
99.	Leuprolide (Lupron)	Reduction in testosterone or estradiol [438]
100.	Liraglutide (Victoza)	Blood glucose & GLP-1 levels [439]
101.	Lixisenatide (Adlyxin)	GLP-1 receptor activation [440]
102.	Loncastuximab tesirine (Zynlonta)	CD19 expression [441]
103.	Lucinactant (Surfaxin)	Improved lung compliance [441]
104.	Luspatercept-aamt (Reblozyl)	Erythroid maturation [441]
105.	Lutropin alfa (Luveris)	LH receptor activation [442]
106.	Margetuximab (Margenza)	HER2 expression [443]
107.	Mecasermin (Increlex)	IGF-1 receptor activation [444]
108.	Menotropins (Menopur)	FSH and LH receptor activation [445]
109.	Mepolizumab (Nucala)	IL-5 neutralization [446]
110.	Metreleptin (Myalept)	Leptin receptor activation [447]
111.	Mirvetuximab Soravtansine	Folate receptor alpha targeting [448]
112.	Mogamulizumab (Poteligeo)	CCR4 targeting [449]
113.	Moxetumomab pasudotox (Lumoxiti)	CD22 expression [450]
114.	Muromonab (Orthoclone OKT3)	CD3 expression [451]
115.	Natalizumab (Tysabri)	α4-integrin saturation [452]
116.	Naxitamab (Danyelza)	GD2 expression [453]
117.	Necitumumab (Portrazza)	EGFR targeting [454]
118.	Nesiritide (Natrecor)	Natriuretic peptide receptor A activation [455,456]
119.	Netakimab (Netakimab)	IL-17A inhibition [457,458]
120.	Nimotuzumab (Theraloc, h-R3)	EGFR targeting [459,460]
121.	Nivolumab (Opdivo)	PD-1 receptor occupancy & T cell function [233]
122.	Nofetumomab fmerpentan (Verluma)	Carcinoembryonic antigen (CEA) targeting [461]
123.	Obiltoxaximab (Anthim)	Protective antigen (PA) binding of Bacillus anthracis [462]
124.	Obinutuzumab (Gazyva)	CD20 targeting [234]
125.	Ocrelizumab (Ocrevus)	CD20 targeting [463]
126.	Ocriplasmin (Jetrea)	Vitreomacular adhesion dissolution [461]
127.	Ofatumumab (Arzerra)	CD20 targeting [235]
128.	Olaratumab (Lartruvo)	PDGFRα phosphorylation levels [376]
129.	Olipudase alfa (Xenpozyme)	Acid sphingomyelinase replacement [464]
130.	Omalizumab (Xolair)	Free serum IgE levels & FcεRI expression on basophils [465]
131.	Oportuzumab monatox (Vicineum)	N-acetylgalactosamine-4-sulfatase targeting [466]
132.	Oprelvekin (Neumega)	Thrombopoietin receptor activation [467]
133.	Oxytocin (Pitocin)	Oxytocin receptor activation [468]
134.	Palifermin (Kepivance)	Keratinocyte growth factor receptor activation [469]
135.	Palivizumab (Synagis)	RSV neutralization in serum [154]
136.	Pancrelipase amylase (Creon)	Pancreatic enzyme replacement [470]
137.	Panitumumab (Vectibix)	EGFR receptor occupancy & phosphorylation [471,472]
138.	Parathyroid/Preotact (Preos)	Parathyroid hormone (PTH) receptor activation [473]
139.	Pegademase bovine (Adagen)	ADA enzyme replacement [474]
140.	Pegaspargase (Oncaspar)	Asparagine depletion [475]
141.	Pegcetacoplan (Empaveli)	Complement C3 inhibition [476]
142.	Peginterferon alfa-2a (Pegasys)	Interferon alpha receptor activation [477]
143.	Peginterferon alfa-2b (PegIntron)	Interferon alpha receptor activation [478]
144.	Pegloticase (Krystexxa)	Uric acid metabolism [479]
145.	Pegvisomant (Somavert)	Growth hormone receptor antagonist [480]
146.	Pembrolizumab (Keytruda)	PD-1 receptor occupancy & PD-L1 expression [481]
147.	Pertuzumab (Perjeta)	HER2 receptor dimerization inhibition [481]
148.	Pertuzumab (Perjeta)	HER2/neu targeting [482]
149.	Pramlintide (Symlin)	Amylin analogue [483]
150.	Protein S human (PROS)	Protein S supplementation [484]
151.	Ramucirumab (Cyramza)	VEGFR2 targeting [485]
152.	Ranibizumab (Lucentis)	VEGF level & macular thickness [486]
153.	Rasburicase (Elitek)	Uric acid conversion to allantoin [487]
154.	Reteplase (Retavase)	Plasminogen activation [488]
155.	Rilonacept (Arcalyst)	Interleukin-1 blockade [489]
156.	Rituximab (Rituxan)	CD20+ B cell depletion [490]
157.	Romiplostim (Nplate)	Thrombopoietin receptor stimulation [491]
158.	Romosozumab (Evenity)	Sclerostin levels & bone mineral density [492]
159.	Sacrosidase (Sucraid)	Sucrase replacement for sucrose digestion [493]
160.	Sargramostim (Leukine)	GM-CSF receptor activation [494]
161.	Sarilumab (Kevzara)	IL-6 receptor blockade & CRP [495]
162.	Sebelipase alfa (Kanuma)	Lysosomal acid lipase replacement [496]
163.	Secretin (SecreFlo)	Secretin receptor activation [497]
164.	Secukinumab (Cosentyx)	PASI score, serum IL-17A levels [241]
165.	Sermorelin (Geref)	GHRH receptor activation [498]
166.	Siltuximab (Sylvant)	IL-6 targeting [499]
167.	Somatotropin (Genotropin)	GH receptor activation [500]
168.	Streptokinase (Streptase)	Plasminogen activation [501]
169.	Tagraxofusp (Elzonris)	CD123-directed cytotoxin [502]
170.	Taliglucerase alfa (Elelyso)	Glucocerebrosidase enzyme replacement [503]
171.	Teduglutide (Gattex)	GLP-2 receptor activation [504]
172.	Tenecteplase (TNKase)	Plasminogen activation [505]
173.	Teriparatide (Forteo)	Bone formation stimulation [472]
174.	Terlipressin (Varpress)	Vasoconstriction through V1 receptor activation [506]
175.	Tesamorelin (Egrifta)	GH-releasing hormone receptor activation [507]
176.	Thymalfasin (Zadaxin)	Immunomodulation, T-cell stimulation [508]
177.	Thyrotropin Alfa (Thyrogen)	Thyroid-stimulating hormone receptor activation [509]
178.	Tirzepatide (Mounjaro)	Dual GLP-1 and GIP receptor agonism [510]
179.	Tisotumab vedotin (Tivdak)	ADC targeting tissue factor [511]
180.	Tocilizumab (Actemra)	IL-6 receptor blockade [512]
181.	Tositumomab (Bexxar)	CD20 targeting [513]
182.	Trastuzumab (Herceptin)	HER2/neu receptor expression [514]
183.	Urofollitropin (Bravelle)	Follicle-stimulating hormone stimulation [515]
184.	Urokinase (Abbokinase)	Plasminogen activation [516]
185.	Ustekinumab (Stelara)	Inhibition of the p40 subunit of interleukin-12 (IL-12) and interleukin-23 (IL-23) [517]
186.	Vasopressin (Vasostrict)	V1 and V2 receptor activation [518]
187.	Vedolizumab (Entyvio)	α4β7 integrin receptor occupancy [519]
188.	Velaglucerase alfa (Vpriv)	Glucocerebrosidase enzyme replacement [520]
